# Engineering probiotics for mucosal vaccination: from the *Escherichia coli* Nissle 1917 paradigm to a translational vaccine platform

**DOI:** 10.3389/fgstr.2026.1980209

**Published:** 2026-10-29

**Authors:** Irene Palenca, Giuseppe Esposito, Marcella Pesce, Giovanni Sarnelli

**Affiliations:** 1 Neuropharmacology and Behavioral Science Laboratory, Santa Lucia Foundation (IRCCS Fondazione Santa Lucia), Rome, Italy; 2 Department of Physiology and Pharmacology “V. Erspamer”, Sapienza University of Rome, Rome, Italy; 3 Nextbiomics S.R.L., Naples, Italy; 4 Department of Clinical Medicine and Surgery, “Federico II” University of Naples, Naples, Italy

**Keywords:** engineered probiotics, *Escherichia coli* Nissle 1917, GALT, intranasal vaccine, mucosal vaccination, oral vaccine, pAIDA, secretory IgA

## Abstract

Mucosal vaccination is attracting renewed interest because many clinically relevant pathogens first encounter the host at gastrointestinal or respiratory surfaces, whereas most established vaccines are administered parenterally and are optimized primarily to induce systemic immunity. Engineered probiotics are emerging as living antigen-delivery vectors that may combine mucosal delivery, transient biological persistence, microbial immune signals, and programmable antigen presentation within a single platform. This strategy is particularly relevant to gastrointestinal vaccination, where access to gut-associated lymphoid tissue (GALT) may support local secretory IgA together with systemic responses. SARS-CoV-2 provides an informative experimental bridge because, despite its dominant respiratory manifestations, it can involve intestinal epithelial cells and gastrointestinal mucosal immunity. Using the pAIDA–Escherichia coli Nissle 1917 (EcN) system as a bounded proof of concept, this Perspective defines an integrated translational framework linking GALT-directed immune priming, effective mucosal antigen dose, genetic stability, chassis de-risking, biocontainment, manufacturing, potency, and post-administration clearance. We explicitly distinguish antigen delivery and immunogenicity from functional immunity, protection, and clinical efficacy, and identify the experiments required to advance probiotic-vectored mucosal vaccines toward clinically testable products.

## Introduction

Mucosal vaccination aims to place immune priming closer to anatomical sites where pathogens are first encountered. Engineered probiotics and other nonpathogenic bacteria can be programmed to produce, secrete, release, or display immunologically active molecules, while non-living approaches such as outer-membrane vesicles provide alternative ways to deliver antigen to mucosal surfaces (1–12). In this Perspective, we use engineered probiotic to denote a probiotic chassis modified to carry a defined vaccine function; probiotic vector to describe that chassis in its delivery role; live bacterial vaccine to describe the final vaccine concept; and live biotherapeutic product (LBP) only in the regulatory context of live microorganisms developed as medicinal products. We retain “immunobiotic” only as a conceptual descriptor and not as a regulatory category.

The gastrointestinal tract is particularly relevant because it is both an infection site and a major immune-inductive organ. Respiratory and gastrointestinal infection are not always biologically separable: respiratory pathogens can engage intestinal mucosa through direct tropism, swallowed material, systemic dissemination, or gut–lung immune crosstalk (13, 14). SARS-CoV-2 is instructive in this regard. Although COVID-19 is dominated clinically by respiratory disease, intestinal epithelial cells express ACE2, gastrointestinal symptoms and fecal viral detection are documented, and intestinal mucosal immune responses participate in the host–virus interaction (15). Thus, SARS-CoV-2 is used here not as a surrogate for classical enteric pathogens but as a model illustrating the continuity of mucosal compartments. The stronger future gastrointestinal test will be direct adaptation to bona fide enteric targets, including enteric viruses such as norovirus and rotavirus and bacterial enteropathogens such as enterotoxigenic Escherichia coli (ETEC) and Shigella, with antigen choice, mucosal correlates of protection, and challenge models defined for each pathogen (4, 13, 16, 17).

The specific contribution of this Perspective is to move beyond the question of whether engineered bacteria can carry vaccine antigens and instead define the integrated design requirements that would allow a probiotic vector to become a controllable mucosal vaccine product. Existing literature describes multiple chassis and delivery formats, but immune design, quantitative antigen exposure, genetic stabilization, ecological containment, pharmaceutical potency, and clearance are often discussed separately. We therefore use our pAIDA–EcN SARS-CoV-2 studies (18, 19) as an experimentally defined case study within a broader comparative landscape, not as a uniquely validated or universally applicable solution. The proposed integrated translational roadmap is summarized in **Figure 1**.

**Figure 1:**
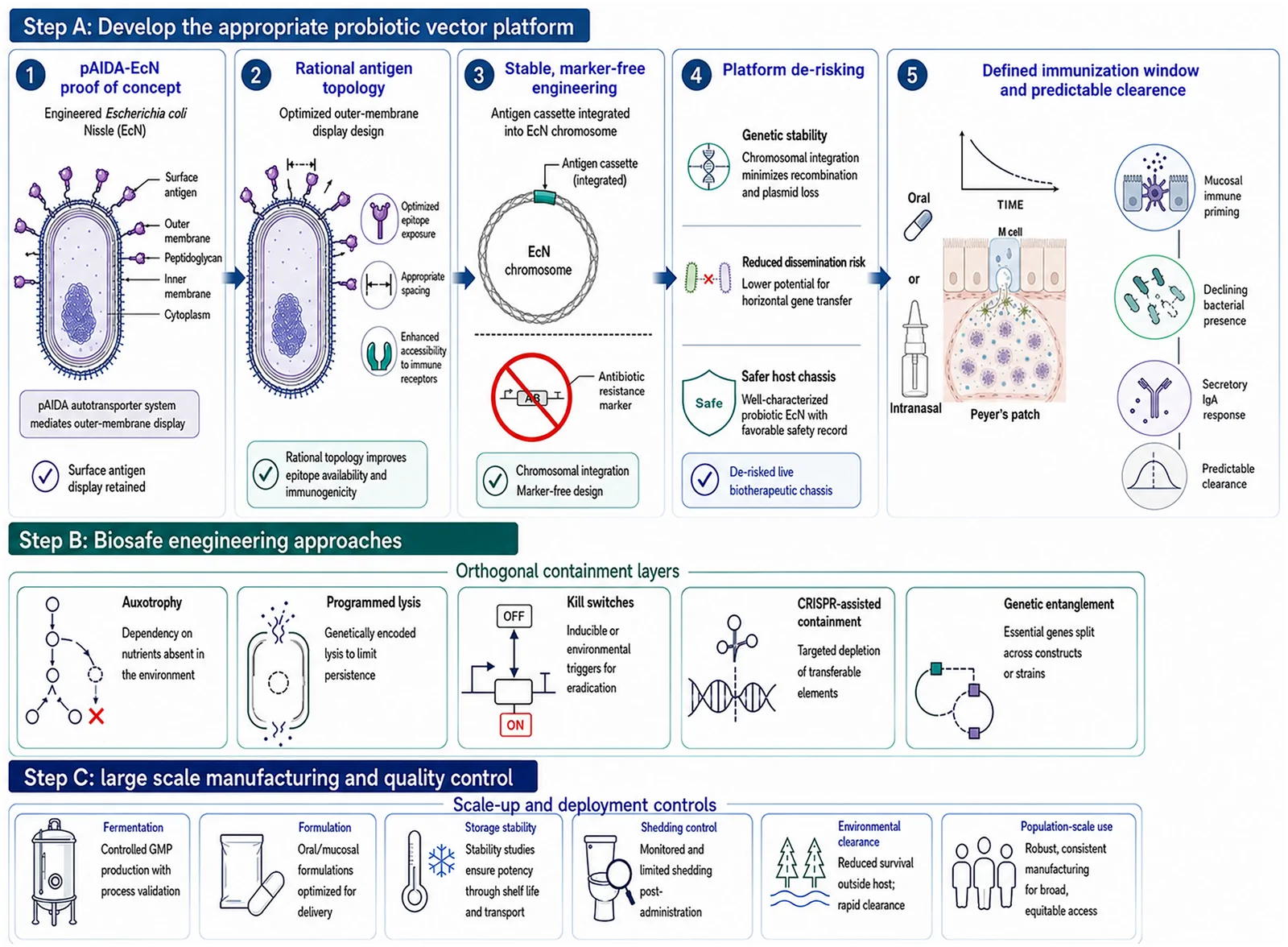
Translational roadmap with explicit evidence-status separation. The pAIDA–EcN surface-display proof of concept is distinguished from preclinical engineering steps that require direct validation and from proposed translational development. AIDA-I is the autotransporter machinery; pAIDA is the corresponding plasmid-based display platform; pAIDA1-SP is the specific SARS-CoV-2 Spike display construct used in the proof-of-concept studies. Future development requires rational antigen topology, chromosomal marker-free engineering, EcN chassis de-risking, route-specific control of persistence, orthogonal biocontainment, quantitative potency testing, and manufacturing and environmental-clearance controls. “Demonstrated” refers only to findings directly supported by the cited pAIDA–EcN studies; downstream stages should not be interpreted as clinically validated.

## Comparative landscape of engineered mucosal vaccine platforms

Engineered bacterial vaccine platforms differ in chassis biology, antigen localization, persistence, and translational maturity. Lactococcus and Lactobacillus systems have been used for mucosal expression or delivery of heterologous antigens, with extensive preclinical experience and, for engineered Lactococcus used as an intestinal protein-delivery vehicle, a human phase I precedent (1, 2, 4, 20, 21). Gram-negative carriers, including probiotic E. coli and Salmonella-derived systems, can support surface display, secretion, lysis-mediated release, or multivalent antigen presentation (22–24). Bifidobacterium-based approaches have also been used to target antigen toward Peyer’s patches and GALT-associated antigen-presenting cells (25). Non-living platforms such as outer-membrane vesicles avoid bacterial replication but do not provide the same capacity for *in situ* antigen production or transient persistence (8, 12). **Table 1** summarizes representative approaches and, importantly, separates measured immunogenicity from evidence of protection and clinical translation. Importantly, no head-to-head comparison between pAIDA–EcN and OMV-based delivery has yet been performed; therefore, no superiority in efficacy or durability is claimed. The comparison is presently conceptual and highlights different product properties rather than demonstrated performance advantages.

**Table 1:** Representative mucosal vaccine platforms and translational evidence framework.

Platform/example	Antigen strategy	Route/model	Immune outcome	Protection evidence	Evidence level/key readout	Main translational limitation/milestone
EcN–pAIDA1-SP (18, 19)	AIDA-I surface display of Spike	Oral and intranasal; mice	Mucosal IgA, systemic IgG; avidity	No live-virus oral challenge; intranasal Spike-injury model only	Proof of concept; quantify antigen-positive fraction and effective mucosal antigen dose	Replace plasmid/markers; demonstrate pathogen protection, containment, potency
Lactobacillus spp (4, 6).	Surface display, secretion or intracellular expression	Mainly oral/intranasal; preclinical infectious-antigen models	Mucosal and systemic responses	Platform- and antigen-dependent	Broad preclinical literature; antigen-specific functional assays	Strain stability, dose standardization, formulation, protection models
Engineered Lactococcus lactis (1, 2, 20, 21)	Secretion/mucosal delivery of heterologous proteins	Oral; preclinical and human intestinal-delivery precedent	Local biological activity; platform-dependent immune responses	Not a general vaccine efficacy precedent	Biocontainment and human phase I precedent for engineered live delivery	Translate containment/manufacturing precedent to antigen-specific vaccine potency
Other E. coli/inducible lysis systems (22, 23)	Surface display, encoded arrays, lysis-mediated release	Oral; preclinical	DC activation, mucosal/systemic cellular responses	Protection shown in selected experimental models	Advanced preclinical; measure lysis kinetics and antigen release	Circuit stability, escape, reproducibility and disease-specific efficacy
Bifidobacterium antigen targeting (25)	Surface functionalization/antigen targeting	Oral; murine tumor immunotherapy	GALT-directed antigen presentation; mucosal/systemic responses	Murine efficacy in specific model	Preclinical; trafficking and APC-targeting readouts	Manufacturing consistency, stability, indication-specific validation
OMV/non-living mucosal platforms (8, 12)	Acellular vesicle-associated antigen delivery	Mainly intranasal; preclinical/selected clinical development	Mucosal IgA and cellular responses	Protection in selected models	Non-replicating comparator; antigen dose is directly measurable	Shorter persistence; formulation/adjuvanticity; no *in situ* antigen production

Within this landscape, AIDA-I is the autotransporter machinery that mediates outer-membrane display; pAIDA refers to the plasmid platform exploiting this AIDA-I display logic; and pAIDA1-SP is the specific construct used to display SARS-CoV-2 Spike on EcN. The transferable principle is therefore not the SARS-CoV-2 construct itself but the assignment of a programmable surface antigenic identity to a biologically active mucosal chassis.

## EcN as a chassis: opportunities and specific safety constraints

EcN is attractive because it survives gastrointestinal transit, interacts with mucus, and provides microbial signals capable of shaping dendritic-cell cytokine responses (26–28). These properties may support antigen delivery in an environment in which immune education is already influenced by commensal and dietary signals. However, parental probiotic use cannot be taken as evidence of safety for an engineered vaccine strain. Genetic modification can alter growth, surface properties, metabolism, persistence, or host interactions and should therefore be followed by phenotypic re-characterization rather than genotype confirmation alone.

EcN also carries genomic features with potential safety implications, most notably the pks/colibactin locus. Deletion of this region has been shown to preserve growth, gastrointestinal fitness, and engineered therapeutic function in preclinical models, supporting chassis de-risking as a rational strategy (29). Such deletion is not automatically neutral, however: the effect on competitive fitness, colonization dynamics, immunostimulation, manufacturing behavior, and vaccine performance should be re-evaluated for each engineered product. The translational goal is therefore not simply a “safer EcN”, but a de-risked chassis whose relevant biological phenotype remains quantitatively characterized after every major engineering step.

## GALT-directed immune priming, oral tolerance, and levels of evidence

Oral vaccination can exploit physiological antigen-sampling pathways within GALT. M cells overlying Peyer’s patches transport luminal material toward subepithelial immune compartments; dendritic cells and other antigen-presenting cells process antigen and provide contextual signals; activated T cells can support B-cell responses, class switching, and differentiation toward IgA-producing plasma cells, while lymphocyte trafficking contributes to dissemination of mucosal immune responses (16, 17, 30). We use “GALT-directed immune priming” as the mechanistic term and retain “teaching the GALT” only as conceptual shorthand for repeated delivery of defined antigenic information to these physiological inductive pathways. These cellular mechanisms are established components of mucosal immunity; what remains hypothetical for pAIDA–EcN is the quantitative contribution of individual sampling routes and whether surface display plus transient persistence improve antigen uptake sufficiently to enhance functional protection.

Repeated intestinal exposure can also promote oral tolerance rather than productive vaccination. Antigen dose, localization, duration, bacterial innate signals, inflammatory context, and dosing frequency can shift the balance between priming and regulatory or hyporesponsive outcomes (10). An engineered probiotic vaccine should therefore be optimized for an immunogenic exposure window rather than maximal persistence or maximal antigen production. This distinction is especially important when moving from proof-of-concept antibody measurements to vaccine efficacy. Antigen delivery, immunogenicity, functional immunity, protection against infection or disease, and clinical efficacy represent sequential but non-equivalent evidence levels. Detection of mucosal IgA or systemic IgG demonstrates immunogenicity; functional neutralization or relevant cellular activity provides stronger mechanistic evidence; protection requires an appropriate pathogen-challenge model; and clinical efficacy ultimately requires human trials.

## Oral and intranasal routes are complementary, not equivalent

In the oral pAIDA–EcN study, repeated administration induced systemic anti-Spike IgG together with antigen-specific IgA in stool, intestinal lavage, and bronchoalveolar lavage, with increasing antibody avidity over time (18). This demonstrates mucosal and systemic immunogenicity but not protection, because no live-virus challenge was performed. In the intranasal study, EcN-pAIDA1-SP induced bronchoalveolar IgA and circulating IgG, and pre-immunized mice showed better preservation of lung morphology and lower inflammatory activation after Spike challenge (19). This was a protein-mediated injury model rather than infection with replicating SARS-CoV-2; it therefore supports functional biological activity in that model, not antiviral protection or clinical efficacy. In the intranasal proof-of-concept study, anti-Spike IgG in serum and IgA in bronchoalveolar lavage fluid were detectable two weeks after the initial administration and reached peak concentrations on day 35 (19). The phrase “long-term immunization” in the original study title therefore refers to the kinetics and persistence of the measured immune response within that experimental observation period and should not be interpreted as evidence of persistence of the bacterial vector. The intestinal residence time of the oral pAIDA–EcN vector was not directly quantified in the proof-of-concept study, because longitudinal vector recovery and clearance were not dedicated endpoints. Accordingly, the duration of the intestinal immunological stimulation period cannot yet be assigned a numerical value, and persistence of antibody responses should not be equated with persistence of the bacterial vector. Future studies should measure vector recovery and clearance in parallel with mucosal and systemic immune kinetics. The integrated translational roadmap is summarized in **Figure 1**.

The two routes impose different constraints. Oral delivery must withstand gastric acidity and bile, interacts directly with the intestinal microbiome, raises questions of oral tolerance and fecal shedding, and primarily targets GALT-associated inductive sites. Intranasal delivery avoids gastrointestinal transit but faces mucociliary clearance, a distinct resident microbiota, and route-specific risks related to local inflammation and administration of live organisms to the respiratory mucosa. Accordingly, dose, persistence, containment, and safety specifications should be route-specific rather than transferred from one mucosal compartment to another.

## From CFU to an effective mucosal antigen dose

For a living vaccine, administered CFU and effective antigen dose are not interchangeable. We therefore propose the concept of an effective mucosal antigen dose: the antigen exposure that is actually available to immune-inductive sites as a combined function of the administered viable dose, the fraction of bacteria expressing or displaying antigen, antigen density per cell, orientation and surface accessibility, expression stability, antigen degradation, bacterial viability, residence time, and access to sampling pathways. This concept provides a quantitative bridge between synthetic-biology design and immunological outcome.

Development should therefore report more than CFU. Useful release and characterization measures include the percentage of antigen-positive bacteria, antigen density or relative surface abundance, batch-to-batch expression stability, genetic retention in the absence of selection, viability after formulation and storage, and persistence at the target mucosa. Where feasible, antigen accessibility should be assessed using conformation- or epitope-sensitive binding assays. These measurements would permit a potency specification that links viable bacteria to biologically relevant antigen presentation rather than treating CFU alone as a sufficient surrogate. Antigen replacement should also not be assumed to be structurally plug-and-play. Substituting Spike with an antigen from a strict enteric pathogen may alter passenger size, folding, oligomerization, linker requirements, export efficiency, epitope orientation, steric accessibility, and achievable surface density. Each new antigen should therefore undergo construct-specific characterization of display geometry, conformational accessibility, expression stability, and retained bacterial fitness before immunological comparisons are interpreted.

## Genetic stability, dissemination, and multilayer biocontainment

The original pAIDA–EcN system was plasmid-based because plasmids are efficient for proof-of-concept engineering. For clinical development, plasmid loss, antibiotic-resistance markers, and mobile DNA are liabilities. Stable chromosomal integration, marker-free editing, defined loci, and controlled promoters can improve product consistency, but chromosomal localization does not eliminate bacterial shedding, release of engineered DNA, or horizontal gene transfer (31–33). Genetic dissemination should therefore be measured directly using recovery of viable engineered strains, qPCR or digital PCR for construct-specific sequences, ex vivo or *in vitro* transfer assays with plausible recipient organisms, and longitudinal sampling of fecal or environmental material. Transfer frequency and persistence of engineered DNA should be reported independently from persistence of viable vaccine bacteria. For pAIDA–EcN specifically, an optimal chromosomal insertion locus has not yet been experimentally established. Rather than designating a locus without comparative data, candidate neutral sites should be screened empirically for absence of polar effects, stable marker-free expression without selective pressure, preservation of growth and mucosal fitness, and maintenance of antigen-display potency. Likewise, a numerical HGT rate has not been measured for the current pAIDA–EcN construct; this remains a required translational endpoint rather than an available property of the platform.

Biocontainment should be multilayered because no single mechanism is failure-proof. Auxotrophy is comparatively simple but can fail when required metabolites are supplied by the host or microbiota. Programmed lysis can couple clearance with antigen release but requires control of timing and escape mutants. Environment-responsive kill switches offer active restriction but add circuit complexity and are vulnerable to mutation. CRISPR-assisted containment can target escaped genetic elements but introduces additional genetic components that themselves require stability testing. Genetic entanglement can achieve very low escape frequencies, although engineering and manufacturing complexity increase (23, 34–36). Programmed lysis, CRISPR-assisted containment, and genetic entanglement have each been experimentally demonstrated in other engineered bacterial systems (23, 35, 36), whereas their implementation as an integrated containment architecture in pAIDA–EcN remains prospective and requires platform-specific validation. The relevant translational metrics are therefore escape frequency, genetic stability over production and storage, function under gastrointestinal conditions, and compatibility with the intended immunization window. For restriction outside the host, the most defensible strategy is therefore orthogonal rather than singular—for example, combining a host-dependent metabolic requirement with an environment-responsive survival circuit and, where appropriate, a DNA-targeting containment layer. However, the optimal combination and its escape frequency have not yet been established for pAIDA–EcN and must be validated experimentally under gastrointestinal, manufacturing, and relevant environmental conditions.

## Manufacturing, potency, regulation, and host variability

A potentially scalable live bacterial vaccine is a medicinal product, not simply a bacterial culture. Critical quality attributes should include strain identity, viable dose, genetic identity and stability, antigen-positive fraction, antigen density or accessibility, purity, absence of undesired contaminants, formulation performance, storage stability, and—where relevant—post-administration shedding. A potency strategy should combine viable-cell enumeration with a product-specific measure linked to mechanism of action, such as quantitative surface-antigen expression and antigen recognition. Manufacturing, formulation, and containment must therefore be co-developed rather than optimized sequentially. For lyophilized or otherwise dried formulations, this should be extended to pre- versus post-processing CFU recovery, residual moisture or water activity, reconstitution kinetics, retention of antigen-positive fraction and surface-antigen accessibility, cassette stability without selection, and real-time or accelerated shelf-life testing under defined temperature and humidity. These are proposed critical quality controls for future product development and were not measured in the original pAIDA–EcN proof-of-concept studies.

Terminology also matters regulatory-wise. A conventional probiotic is not equivalent to an LBP, and an engineered live vaccine presents additional GMO and vaccine-specific considerations. European frameworks for LBPs emphasize quality, safety, and efficacy requirements for medicinal products containing live microorganisms (37), while recombinant vaccine strains additionally raise environmental-release and genetic-containment questions. Relevant enabling approaches include autotransporter display, engineered-probiotic vaccination, and needle-free delivery (38–40). Claims of scalability should therefore remain conditional: fermentation can be intrinsically scalable, but population-level deployment is only plausible if viability, antigen potency, containment, formulation, storage, and regulatory consistency are demonstrated.

Human reproducibility will also depend on host and microbiome variability. Diet, antibiotics, age, inflammation, immune status, and baseline microbiome composition may alter bacterial survival, antigen exposure, and clearance (32, 33). These variables should be incorporated into translational study design, including stratification, recovery of administered strains, mucosal pharmacokinetic-like measurements, and predefined analyses of factors associated with response heterogeneity. The magnitude of these effects has not been quantified specifically for pAIDA–EcN; consequently, age-, diet-, inflammation-, antibiotic-, and microbiome-dependent changes in persistence or antigen exposure should be regarded as testable sources of variability rather than established effect sizes for this platform.

## Discussion: priorities for clinical translation

The central perspective is that engineered probiotic vaccination should be developed as an integrated immunological, genetic, ecological, and pharmaceutical system. The pAIDA–EcN studies establish a bounded proof of concept: a probiotic chassis can be assigned a surface antigenic identity and can induce antigen-specific mucosal and systemic immune responses after oral or intranasal administration (18, 19). They do not establish protection against replicating SARS-CoV-2, efficacy against enteric pathogens, or clinical readiness.

The next development stage should therefore be judged against a sequence of increasingly stringent milestones: reproducible antigen presentation; defined effective mucosal antigen dose; GALT-directed immune priming without dominant tolerance; functional immune activity; protection in pathogen-challenge models; genetic and phenotypic stability; controlled persistence and environmental clearance; and a validated manufacturing/potency package. Future gastrointestinal studies should prioritize bona fide enteric targets and directly test whether the platform adds value over alternative bacterial and non-living mucosal delivery systems. In that framework, “teaching the GALT” remains a useful conceptual metaphor, but the experimentally testable objective is precise GALT-directed immune priming within a predefined immunization window. Several of the most important translational quantities therefore remain intentionally unresolved: the optimal EcN chromosomal insertion site, the numerical relationship between administered CFU and mucosally available antigen, the duration of vector persistence, the HGT frequency, and comparative efficacy versus non-living carriers. We regard these not as assumptions to be filled by extrapolation, but as explicit experimental milestones that should be measured before clinical translation is claimed.

This shift—from demonstrating that an engineered probiotic can display an antigen to defining how much functional antigen reaches immune-inductive sites, how protection is proven, and how the organism is predictably contained—constitutes the main translational contribution of this Perspective. It also provides a practical framework for deciding when an interesting synthetic-biology construct has matured into a clinically testable mucosal vaccine candidate.

## Data Availability

The original contributions presented in the study are included in the article/supplementary material. Further inquiries can be directed to the corresponding authors.
